# A Rare Case of De Garengeot Hernia With Acute Appendicitis: Diagnostic and Surgical Challenges in a Regional Setting

**DOI:** 10.7759/cureus.98590

**Published:** 2025-12-06

**Authors:** Isabelle Huynh, Paul Strauss

**Affiliations:** 1 General Surgery, Central Gippsland Health, Gippsland, AUS

**Keywords:** atypical appendicitis, de garengeot's hernia, open and laparoscopic surgery, regional hospital, surgical repair of hernia

## Abstract

Appendicitis and incarcerated groin hernias are common surgical emergencies, typically managed independently. A De Garengeot hernia, defined as a femoral hernia containing the vermiform appendix, represents a rare convergence of both pathologies and poses unique diagnostic and operative challenges, particularly in resource-limited, regional settings.

We present a case of a patient who arrived at a regional hospital with a painful, irreducible right-sided groin lump. CT imaging revealed a De Garengeot hernia with associated acute appendicitis. The patient underwent laparoscopic appendicectomy followed by open femoral hernia repair using suture technique without mesh, due to intraoperative evidence of inflammation and contamination.

A hernia diagnosis is often clinical, but imaging is often used in stable patients to clarify anatomy and guide management. Surgical decision-making must consider contamination risk, with mesh repair avoided in infected fields to minimise complications such as mesh infection and recurrence.

This case highlights the importance of early imaging, individualised surgical planning, and flexibility in operative approach when managing rare pathologies like De Garengeot hernia with acute appendicitis in regional environments. Awareness of this entity and appropriate surgical strategy can lead to successful outcomes, even in resource-limited settings.

## Introduction

A De Garengeot hernia with acute appendicitis, defined as a femoral hernia that contains an inflamed appendix, is a rare but important clinical entity. Both appendicitis and incarcerated groin hernias are common general surgical emergencies. The unusual presentation of a De Garengeot hernia alters the typical management of appendicitis and incarcerated groin hernias, as it necessitates both appendicectomy and hernia repair, often in an acutely inflamed and anatomically constrained environment.

For appendicitis, laparoscopic appendicectomy has surpassed open appendicectomy in usage. Some patients with perforated appendicitis may benefit from initial antibiotic therapy followed by interval appendicectomy, and several trials have even suggested that it is feasible to treat uncomplicated appendicitis non-operatively with antibiotics alone [1]. For incarcerated groin hernias, mesh repair reduces recurrence without an increase in postoperative complications and should be considered in clean cases. However, in the setting of bowel resection, mesh repair might increase the incidence of surgical site infection [2].

In this case report, we present the surgical management at a regional hospital of a patient with a De Garengeot hernia complicated by acute appendicitis and discuss the considerations and implications of hernia repair in the setting of acute intra-sac inflammation.

## Case presentation

A 73-year-old female presented to a regional hospital at midnight with a few hours' history of sudden-onset right-sided lower abdominal pain associated with nausea. Her past medical history included two previous cerebrovascular accidents, hypertension, and a conservatively managed hiatus hernia. She was taking aspirin, esomeprazole, candesartan, and desvenlafaxine. A recent uncomplicated surveillance colonoscopy had been performed due to a family history of colorectal cancer on her maternal side.

On clinical examination, she was haemodynamically stable. Her abdomen was soft and non-distended but revealed marked tenderness with an irreducible mass in the right inguinal region. Laboratory investigations demonstrated a normal white cell count (WCC 3.0 x10⁹/L) and C-reactive protein (CRP <1 mg/L). Other biochemical parameters were also within normal limits.

**Figure 1:**
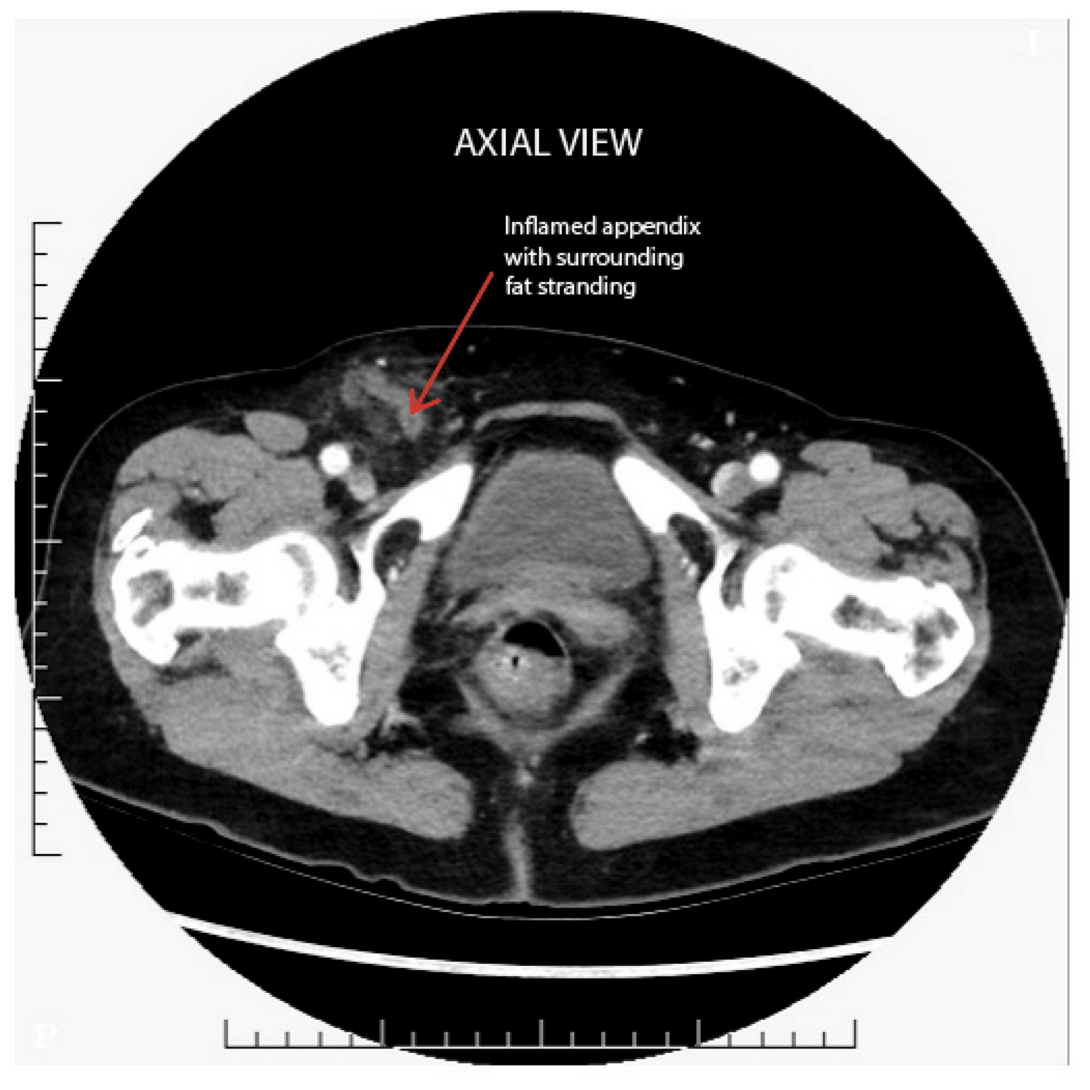
CT axial view of De Garengeot hernia with inflamed appendix and surrounding fat stranding

**Figure 2:**
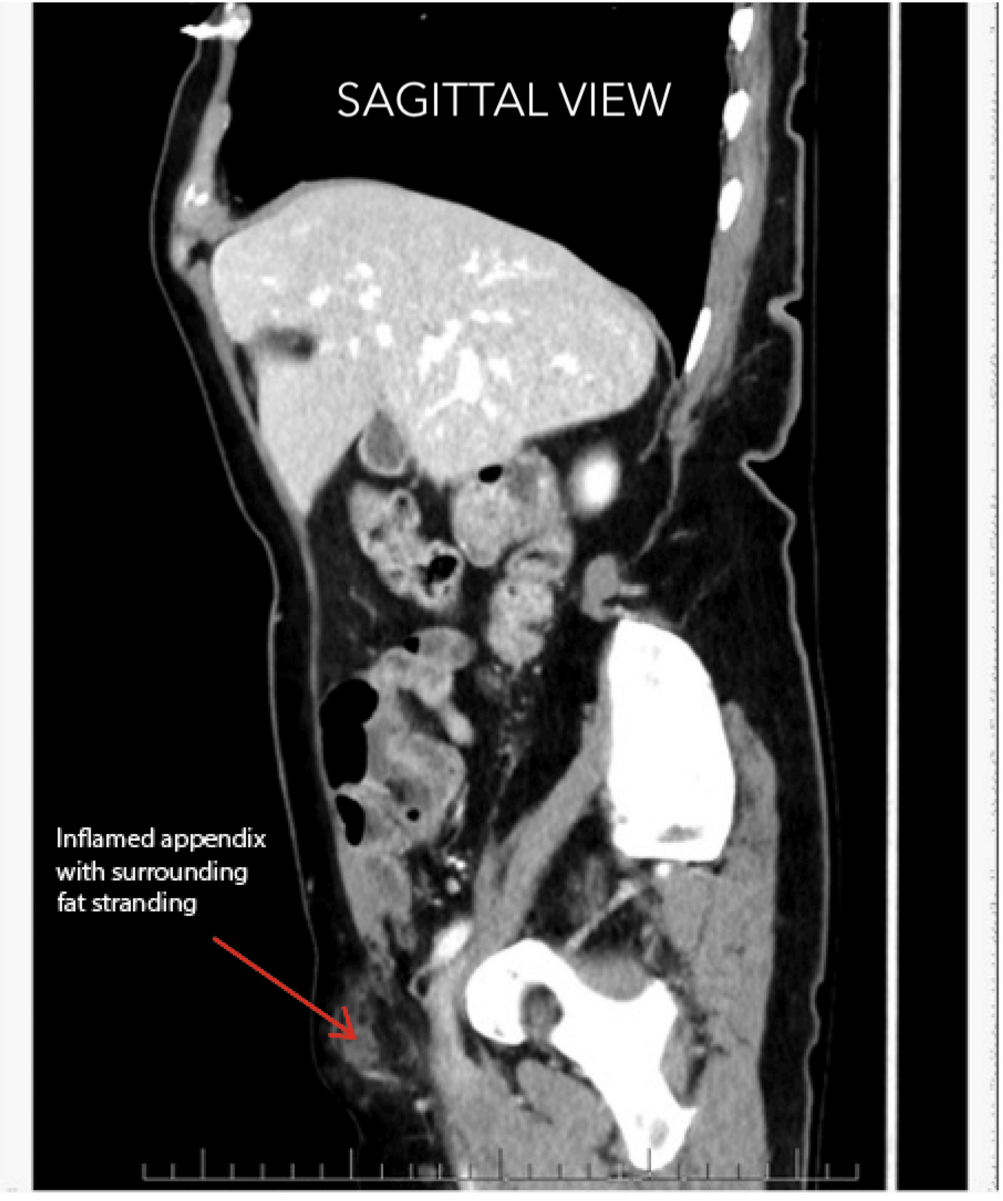
CT sagittal view showing inflamed appendix and fat stranding in De Garengeot hernia

An emergency CT of the abdomen and pelvis (CTAP), Figures 1, 2, showed a right-sided femoral hernia with a 10 mm defect. However, it was reported as a right-sided indirect inguinal hernia. The hernial sac appeared to contain the appendix, with surrounding inflammatory changes suggestive of early appendicitis.

Given the patient’s stable clinical condition and reassuring biomarkers, surgery was deferred until the following morning. Another factor for deferring to the morning was due to the regional nature of the hospital and limited resources, such as out-of-hours theatres, unless deemed clinically relevant. A transverse incision was made below and parallel to the right inguinal ligament. Intraoperatively, the hernial sac was identified within the femoral canal. Careful dissection revealed an inflamed appendix with a necrotic tip incarcerated within the hernia.

**Figure 3:**
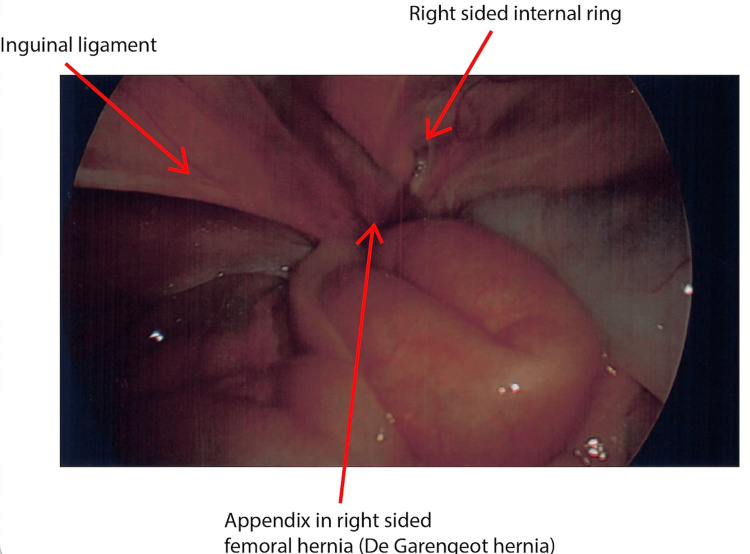
Laparoscopic view of De Garengeot hernia

**Figure 4:**
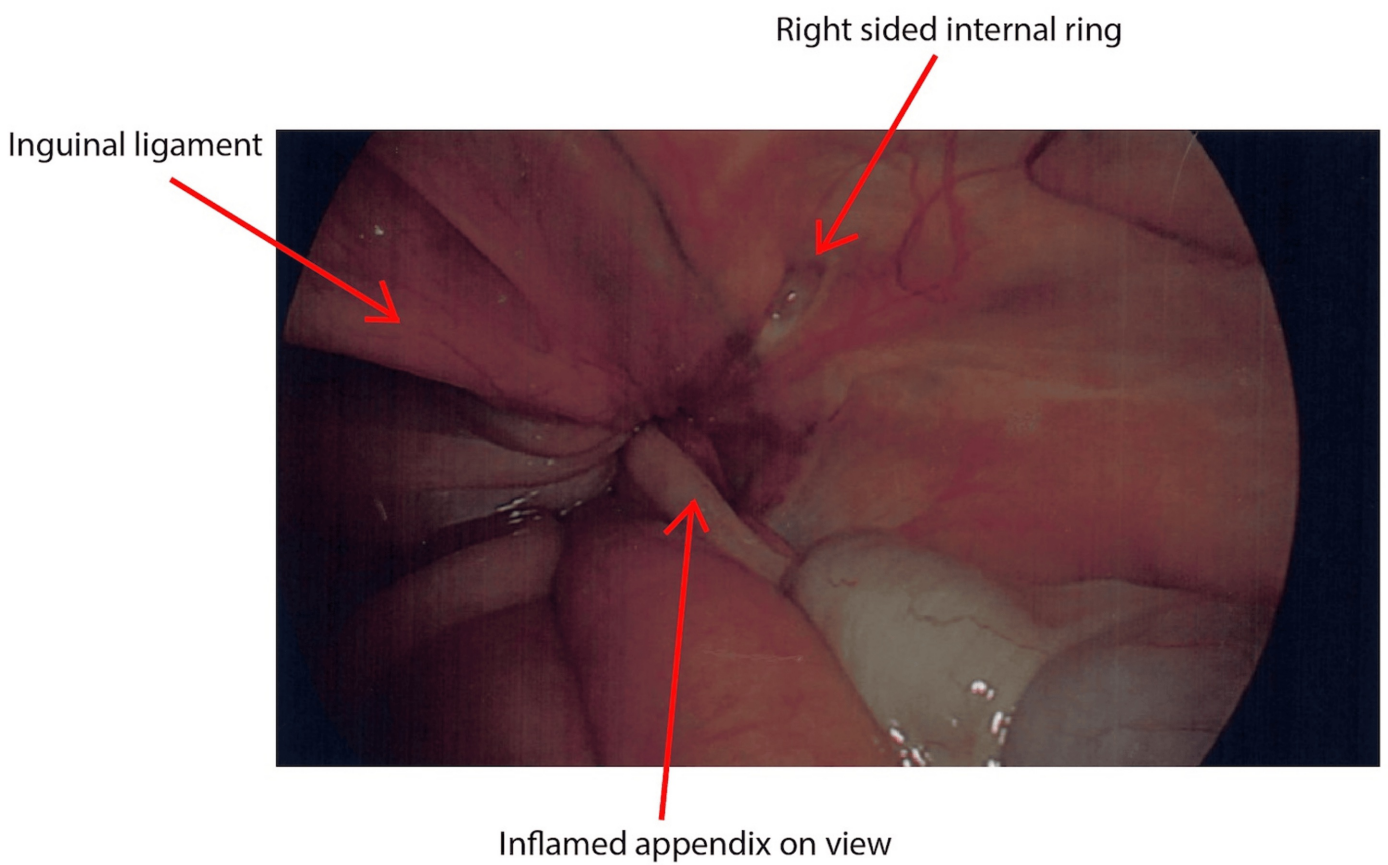
Laparoscopic view with appendix on view

The decision was made to convert to a laparoscopic approach to facilitate a safe and controlled appendicectomy. The appendix was seen medial to the right internal ring and below the inguinal ligament (Figures 3, 4). The appendix was gently reduced from the femoral canal, followed by dissection of the mesoappendix and completion of the appendicectomy. The laparoscopic ports and umbilical fascia were closed appropriately.

The femoral hernia was then re-examined. Peritoneal fat was excised, and a primary suture repair was performed without mesh due to the risk of infection in the setting of contaminated fields.

Post-operatively, the patient recovered well and was discharged the following day with appropriate safety netting. She was subsequently reviewed in the outpatient clinic and discharged from surgical follow-up. Histopathology also confirmed acute appendicitis.

## Discussion

Femoral hernias occur as a result of weakening and protrusion of abdominal viscera through the femoral canal, bordered superiorly by the inguinal ligament and laterally by the femoral vessels [3]. The sac may contain preperitoneal fat, omentum, small bowel, or other structures [4]. Femoral hernias are rare, occurring in about 3-4% of all groin hernias. A De Garengeot hernia, which is a femoral hernia containing the appendix, is even rarer, occurring in only 0.5-1% of femoral hernia cases [5]. The occurrence of appendicitis within this type of hernia is even more rare, with an estimated incidence of 0.08% to 0.13% [6]. The patient may present with symptoms of appendicitis combined with symptoms of an incarcerated hernia with a bulge that is irreducible, similar to the case presented above. In a systematic review in 2022 of published case reports of De Garengeot hernia with acute appendicitis, 83 reports were published with 111 cases identified [7].

Both incarcerated groin hernias and appendicitis are often diagnosed clinically based on the patient’s history, examination, and biochemical markers. In contrast to this patient, a systematic review found that elderly patients with complicated appendicitis often presented with higher WBC, CRP, and total bilirubin and lower lymphocyte levels [8]. Imaging is preferred if the patient is clinically stable prior to any planned operation. This is to ensure the correct diagnosis is found and the surgical planning is optimised. Imaging helps with locating the appendix and locating any collections, which may mean a planned delay in surgery or that the diagnosis is completely incorrect. It prevents unnecessary harm to patients but also helps with assessing the correct treatment pathway.

In this patient, two critical diagnoses were found, which resulted in laparoscopic appendicectomy and open suture repair of the femoral hernia. In our case, the initial examination and CT report were different from the intraoperative findings. The CT was reported to be a right-sided indirect inguinal hernia with an appendix in situ, by definition, an Amyand hernia. Intraoperatively, the hernia was found to be a femoral hernia with an appendix in situ, a De Garengeot hernia. As per Figures 3, 4, the appendix was seen medial to the right internal ring and below the inguinal ligament. This highlights the importance of critical examination, reviewing images, and ensuring that intraoperative findings are accurately described. Both types of hernia are rare, and those hernias associated with appendicitis, perforation, or abscess are even scarcer presentations. The treatment of Amyand's hernia and De Garengeot's hernia is not standardised. Generally, hernia repair is performed, but disagreement remains regarding the use of mesh and performing an appendectomy [9].

An open approach was initially undertaken to assess for any overt contamination or perforation. Dissection of the femoral hernial sac revealed an appendix with a necrotic tip. Given this finding, the procedure was converted to a laparoscopic approach to allow thorough visualisation of the intra-abdominal cavity and to safely complete the appendicectomy. After the appendix was isolated and removed, a copious peritoneal washout was performed.

Subsequently, an open femoral hernia repair was carried out using a primary suture technique. Mesh was deliberately avoided due to the high risk of infection in the context of local inflammation and contamination. Placement of prosthetic mesh in this setting is associated with increased risk of complications, including surgical site infection, mesh infection, and hernia recurrence. The most common reported complication of de Garengeot hernia repair is wound infection, reported to occur in up to 29% of patients [5]. However, in cases where the appendix is non-inflamed or once any acute inflammation has resolved, mesh repair may be considered as a viable option.

A systematic review on De Garengeot hernia with acute appendicitis showed the most common surgical approach was the low approach, 35 (31%), followed by the inguinal approach, 23 (21%). A total of 81 (72%) underwent herniorrhaphy with non-absorbable sutures, and 20 had mesh repairs (18%). Ten (9%) patients were reported to have postoperative morbidity, with wound infection being the most common complication and one recorded death [7]. Another technique was reported showing minimally invasive management of De Garengeot hernia with staged robotic hernia repair [10].

## Conclusions

Appendicitis and incarcerated groin hernias are common surgical emergencies when presenting in isolation. However, the concurrent presentation of both conditions, as seen in a De Garengeot hernia with appendicitis, a femoral hernia containing an inflamed appendix, is rare but surgically treatable. Management of this condition requires careful consideration of intraoperative findings, particularly the degree of contamination and inflammation, which significantly influences the risk of mesh-related complications.

The choice of treatment modality often depends on surgeon preference, guided by clinical judgement and intra-operative assessment. In cases where contamination is evident, mesh use is generally avoided due to the increased risk of infection. Conversely, if the appendix is non-inflamed or contamination is minimal, mesh repair may be safely employed to reduce the risk of hernia recurrence.
